# Finding the Missing *IMP* Gene: Overcoming the Imipenemase *IMP* Gene Drop-Out in Automated Molecular Testing for Carbapenem-Resistant Bacteria Circulating in Latin America

**DOI:** 10.3390/antibiotics14080772

**Published:** 2025-07-30

**Authors:** Jose Arturo Molina-Mora, Ángel Rojas-Varela, Christopher Martínez-Arana, Lucia Portilla-Victor, Isaac Quirós-Fallas, Miryana Sánchez-Fonseca, Xavier Araya, Daniel Cascante-Serrano, Elvira Segura-Retana, Carlos Espinoza-Solís, María Jose Uribe-Calvo, Vanessa Villalobos-Alfaro, Heylin Estrada-Murillo, Stephanie Montoya-Madriz, Warren Madrigal, Mauricio Lizano, Stefany Lozada-Alvarado, Mariela Alvarado-Rodríguez, Mauricio Bolaños-Muñoz, Cristina García-Marín, Javier Alfaro-Camacho, Gian Carlo González-Carballo, Leana Quirós-Rojas, Joseph Sánchez-Fernández, Carolina Chaves-Ulate, Fernando García-Santamaría

**Affiliations:** 1 Centro de Investigación en Enfermedades Tropicales & Facultad de Microbiología, Universidad de Costa Rica, San José 11501-2060, Costa Ricachristopher.martinez@ucr.ac.cr (C.M.-A.); miryana.sanchez@ucr.ac.cr (M.S.-F.); fernando.garcia@ucr.ac.cr (F.G.-S.); 2 Caja Costarricense del Seguro Social, San José 10105-1000, Costa Ricagcgonzal@ccss.sa.cr (G.C.G.-C.);; 3 Laboratorio Clínico y Banco de Sangre, Universidad de Costa Rica, San José 11501-2060, Costa Rica; betty.lozada@ucr.ac.cr (S.L.-A.);

**Keywords:** carbapenems, tailored qPCR, gene drop-out, IMP, Costa Rica, Latin America, inconsistent laboratory results, whole genome sequencing, antimicrobial resistance

## Abstract

Carbapenem resistance is considered one of the greatest current threats to public health, particularly in the management of infections in clinical settings. Carbapenem resistance in bacteria is mainly due to mechanisms such as the production of carbapenemases (such as the imipenemase IMP, or other enzymes like VIM, NDM, and KPC), that can be detected by several laboratory tests, including immunochromatography and automated real-time PCR (qPCR). **Methods**: As part of local studies to monitor carbapenem-resistant bacteria in Costa Rica, two cases were initially identified with inconsistent IMP detection results. A possible gene drop-out in the automated qPCR test was suggested based on the negative result, contrasting with the positive result by immunochromatography and whole-genome sequencing. We hypothesized that molecular testing could be optimized through the development of tailored assays to improve the detection of *IMP* genes. Thus, using *IMP* gene sequences from the local isolates and regional sequences in databases, primers were redesigned to extend the detection of *IMP* alleles of regional relevance. **Results**: The tailored qPCR was applied to a local collection of 119 carbapenem-resistant isolates. The genomes of all 14 positive cases were sequenced, verifying the results of the custom qPCR, despite the negative results of the automated testing. **Conclusions**: Guided by whole-genome sequencing, it was possible to extend the molecular detection of *IMP* alleles circulating in Latin America using a tailored qPCR to overcome *IMP* gene drop-out and false-negative results in an automated qPCR.

## 1. Introduction

The monitoring and study of antibiotic resistance is a fundamental strategy for detecting, characterizing, and tracking phenotypic profiles and antimicrobial resistance mechanisms in bacteria [1]. This strengthens therapeutic decision-making at both the clinical and epidemiological surveillance levels, and is one of the most important diagnostic stewardship interventions in hospital settings [2]. According to the World Health Organization (WHO), the priority (critical or high) approach to the problem of antibiotic resistance includes bacterial groups such as *Acinetobacter baumannii*, Enterobacterales, and *Pseudomonas aeruginosa* resistant to carbapenems [3,4]. Carbapenems (such as imipenem, meropenem, doripenem, and ertapenem) are a class of broad-spectrum β-lactam antibiotics and are considered last-resort drugs for the treatment of severe infections caused by multidrug-resistant Gram-negative bacteria [5]. Therefore, carbapenem resistance is considered one of the greatest current threats to public health, particularly in the management of infections in clinical settings [6,7].

Carbapenem resistance in bacteria is mainly due to mechanisms that include the production of carbapenemases (β-lactamase enzymes that degrade these antibiotics), decreased permeability to prevent antibiotic entry [2,8], overexpression of efflux pumps [5], and reduction in the antibiotic’s affinity for its site of action [9].

In the case of *bla* genes, these are responsible for the production of β-lactamase enzymes [10]. These genes encode β-lactamases capable of inactivating carbapenems, such as class A enzymes (e.g., *KPC* gene), class B metallo-β-lactamases with genes such as *NDM*, *VIM*, and *IMP*, as well as class D oxacillinases (e.g., *OXA-48-like*) [11]. These genes have been identified in Gram-negative bacteria and are often located on plasmids or integrons, facilitating their transmission across different species [12,13].

Thus, rapid and accurate detection of carbapenem-resistant bacteria is essential in a diagnostic stewardship approach for infection treatment and controlling their spread [10,14,15]. Clinically, there are laboratory tests available for the detection of carbapenemases after bacterial identification and antibiotic susceptibility testing. These tests include technologies such as immunochromatography using monoclonal antibodies directed against specific epitopes in the protein [8,14], and automated real-time PCR (qPCR), a sensitive and specific test commonly used to confirm the presence of genes [10,15].

However, the performance of these tests depends entirely on their design. In PCR tests, the phenomenon known as gene drop-out can occur when mutations or variations arise in the target sequences where primers or probes bind, leading to false-negative results, despite no inhibition of the PCR reaction [16,17,18]. In the case of immunochromatographic tests, their performance can also be affected by single-point variants [14]. Genomic variations affecting one test do not necessarily impact the other (since each targets different sites), which can lead to inconsistent results between tests [8,14]. Verification and clarification of such inconsistencies are resolved with whole-genome sequencing studies to confirm the presence or absence of the gene of interest [16,19].

Automated qPCR systems typically use primers designed based on genome database information [19,20]. Due to the limited reporting of bacterial genome sequences circulating in regions such as Latin America [21], even for high-priority antimicrobial-resistant pathogens, the performance of such molecular strategies could be affected by gene alleles not considered during design.

As part of local and regional efforts to address carbapenem resistance and enhance representation in genome databases, various studies have analyzed complete genomes of pathogens circulating in Latin America [9,22,23,24,25,26]. In Costa Rica, recent studies have led to the generation of a collection of carbapenem-resistant clinical Gram-negative isolates [27]. As part of this initiative, in 2024, two cases were identified with inconsistent IMP detection results between immunochromatographic and automated qPCR tests, which motivated this study. Discrepancies with other resistance genes have not been reported. This phenomenon was further investigated using previously sequenced cases and the isolate collection (119 strains). We hypothesized that molecular testing could be optimized through the development of tailored assays to improve the detection of *IMP* genes. All strains were screened with a tailored qPCR test to detect *IMP*, and the positive cases were validated via whole-genome sequencing. Thus, the aim of this study was to extend the molecular detection of IMP alleles by a tailored qPCR, guided by whole-genome sequencing, in carbapenem-resistant isolates circulating in Latin America to overcome *IMP* gene drop-out and false-negative results in an automated qPCR system.

## 2. Materials and Methods

**Figure 1:**
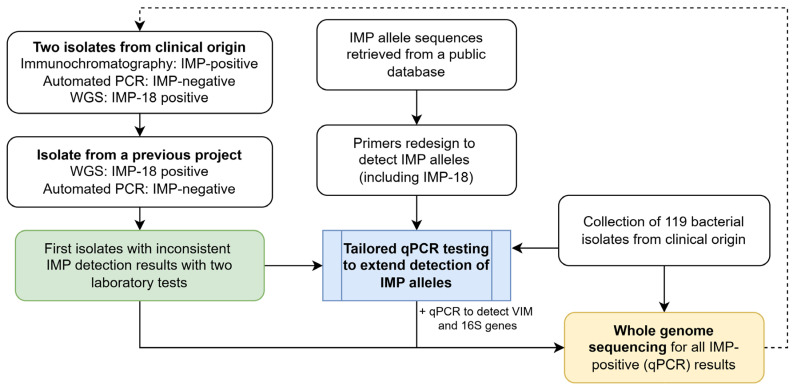
Overview of the study workflow. Extending the detection of *IMP* alleles prompted by inconsistent results between two laboratory tests. Tailored qPCR was used to assess 119 clinical isolates, followed by validation through whole-genome sequencing for *IMP*-positive cases.

This study addressed inconsistencies between laboratory test results and a tailored molecular assay that was assessed to extend the detection of *IMP* genes in clinically derived bacterial strains, including isolates from Costa Rica that were not initially detected by an automated qPCR system. Whole-genome sequencing validated the results. The overall strategy implemented in this study is summarized in Figure 1.

### 2.1. Bacterial Isolates

The bacterial isolates used in this study are part of a collection of bacterial isolates (119 carbapenem-resistant strains) maintained at the University of Costa Rica (Costa Rica). These isolates were sent from various hospitals and healthcare centers across the country for specialized analysis by molecular biology and sequencing techniques.

Inconsistent results between laboratory tests (automated qPCR—see below—and the immunochromatography NG-Test CARBA 5, NG Biotech, Guipry-Messac, France) were initially reported in 2 cases (Table 1), and an additional case was added from a previous project, for strain *P. aeruginosa* AG1 (see Section 3). Ethical review and approval were not required for this study, as no human participants were involved, in accordance with local legislation and institutional policies. This study was approved under document VI-8257-2023 by Vicerrectoría de Investigación, Universidad de Costa Rica, Costa Rica.

### 2.2. Identification and Antimicrobial Susceptibility Testing

Clinical bacterial isolates were subjected to species-level identification and antimicrobial susceptibility testing using the VITEK^®^ 2 automated system (bioMérieux, Marcy-l’Étoile, France), following the manufacturer’s instructions with cards for Gram-negative bacilli. The minimum inhibitory concentrations (MICs) were determined by the system, and results were interpreted according to the Clinical and Laboratory Standards Institute guidelines.

### 2.3. Automated qPCR Analysis for Carbapenemase Gene Detection

Carbapenemase gene detection by qPCR was performed using the GeneXpert^®^ system (Cepheid, Sunnyvale, CA, USA) with the Xpert^®^ Carba-R assay, following the manufacturer’s instructions for the identification of carbapenemase genes, including *KPC*, *NDM*, *VIM*, *OXA-48*, and *IMP*. Results were interpreted automatically by the system’s software.

### 2.4. Genomic DNA Extraction and Quantification

Genomic DNA was extracted from bacterial isolates using the NucleoSpin^®^ Tissue kit (Macherey-Nagel, Düren, Germany) according to the manufacturer’s instructions for bacteria, with a final elution in 100 μL of elution buffer. DNA concentration was measured using the Qubit™ 4 Fluorometer (Thermo Fisher Scientific, Waltham, MA, USA), and DNA purity was assessed spectrophotometrically using a NanoDrop™ 2000 (Thermo Fisher Scientific).

### 2.5. Redesign of Primers to Extend Detection of IMP Alleles

The genomic sequences of the *IMP* gene alleles were downloaded from the ResFinder database [28]. All sequences were used for primer design using the Primer-BLAST platform (https://www.ncbi.nlm.nih.gov/tools/primer-blast/ accessed on 28 March 2025) [29]. Final primer selection (Table 2) was based on the identification of conserved regions through the comparison of different allele sequences, after a multiple sequence alignment performed with MAFFT [30]. Special emphasis was placed on alleles circulating in Latin America that, according to preliminary analyses, showed inconsistent results or were not expected to be amplified in the automated qPCR assay, as well as those known to be successfully amplified by this system [31,32]. Selected primers bind gene regions that we used previously for molecular testing [33].

In addition, due to the potential co-occurrence of *VIM* and *IMP* genes, a complementary qPCR assay was implemented to detect the *VIM* gene based on previous work [34]. The 16S rRNA housekeeping gene was also included as an internal control to validate negative results for carbapenemase genes (Table 2), based on primers by [35].

### 2.6. Tailored qPCR

Conditions for qPCR were standardized for *IMP*, *VIM*, and 16S rRNA genes, similar to our previous work [36]. Each qPCR reaction consisted of 12.5 μL of SYBR Green Master Mix (Thermo Scientific™, Waltham, MA, USA), 10 μL of PCR-grade water, 0.25 μL of each primer (Table 2), and 2 μL of DNA template.

Amplification was performed using a Rotor-Gene Q Real-Time PCR cycler (QIAGEN, Hilden, Germany) under the following thermocycling conditions for *IMP* and 16S genes: initial denaturation at 95 °C for 5 min, followed by 30 cycles of 95 °C for 15 s, 48 °C for 30 s, and 72 °C for 30 s. The final stage included 48 °C for 7 min. For the *VIM* gene, the protocol was the same but the annealing temperature was adjusted to 60 °C. After amplification, a melting curve analysis was performed from 60 to 95 °C, with 0.5 °C increments, to assess amplicon specificity and detect potential non-specific products. Appropriate controls were included for each case. Once optimal amplification conditions were established, final assays were performed with the three genes for all 119 isolates. Ct values were calculated based on a threshold of 0.100 for all cases.

### 2.7. Whole-Genome Sequencing, Assembly, and Annotation

Genomic DNA was sequenced using Illumina technology (Illumina Inc., San Diego, CA, USA) at Novogene, Sacramento, CA, USA (https://www.novogene.com/). The sequencing library was prepared using a standard Illumina DNA shotgun library preparation protocol, and paired-end reads of 150 bp were generated using a NovaSeq X Plus Series (Illumina, San Diego, CA, USA), resulting in approximately 1.00 Gb of raw data per sample.

Bioinformatic analyses were performed using the HPC-UCR computational cluster (Universidad de Costa Rica, https://hpc.ucr.ac.cr/ (accessed on 28 March 2025)). Raw files were evaluated using FastQC v0.12.1 [37], and Trimmomatic v0.38 [38] for trimming (Q > 30). For de novo genome assembly, Megahit v1.2.9 [39] and Unicycler v0.5.1 [40] were used, considering contigs with a minimum length of 1000 bp. The best assembly was selected based on the 3C criterion [41,42]. Structural annotation was achieved with Prokka v1.14.6 [43]. Genome functional annotation was focused on antimicrobial resistance genes using Abricate v1.0.1 (https://github.com/tseemann/abricate (accessed on 28 March 2025)), and genotyping by multilocus sequence typing using MLST v2.0 (https://cge.food.dtu.dk/services/MLST/ (accessed on 28 March 2025)) within the PathogenWatch platform (https://pathogen.watch/ (accessed on 28 March 2025)).

## 3. Results

This study investigated discrepancies in laboratory test results and focused on the implementation of a tailored qPCR assay to extend the detection of *IMP* genes in clinically derived bacterial isolates, including strains with a preliminary negative result in an automated qPCR system (Figure 1). Initially, two cases from 2024 with discordant laboratory test results were identified. These presented positive results for the IMP protein by immunochromatography but failed to detect the corresponding gene using an automated qPCR system. Whole-genome sequencing analysis of these isolates confirmed the presence of the *IMP-18* allele. The inability of the automated system to detect the *IMP-18* gene was further verified using another IMP-18-positive isolate, *P. aeruginosa* AG1, which was originally isolated in Costa Rica in 2010 and has been extensively studied at multiple omics levels over the past 15 years [23,33,44,45]. Based on the results of the genomic context and transcriptomic expression of the *IMP-18* gene, this strain was used as a positive control for IMP, while strain *P. aeruginosa* PAO1 (known as IMP-negative) was used as a negative control. All the initial inconsistent results are shown in Table 1.

**Figure 2:**
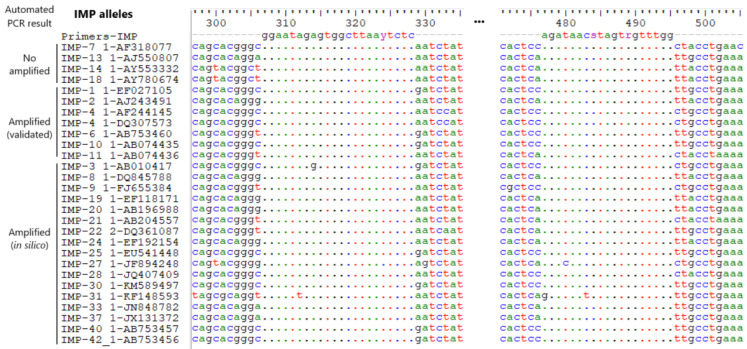
Sequence alignment of *IMP* gene amplicons targeted by redesigned qPCR primers, covering alleles not detected by the automated qPCR. Primer-binding regions are shown in the first row; dots indicate conserved sites. *IMP* alleles are categorized by amplification outcome in the automated qPCR: not amplified, experimentally validated, or in silico predicted.

Considering these observations and reports of *IMP-18*–harboring strains in other regions of Latin America, a redesign of primers was undertaken to extend the detection of *IMP*, including *IMP-18* and other alleles (Table 2 and Figure 2). This stage involved an exhaustive review of available *IMP* gene sequences and alleles in public databases. The primer design aimed to include alleles not detected by the automated qPCR system (alleles 7, 13, 14, and 18) as well as those that were successfully amplified, either experimentally validated or predicted based on manufacturer data. *VIM* and 16S rRNA genes were also included as target and internal control genes, respectively (Table 2).

Once the new primers were acquired, the qPCR-based screening was performed on 119 clinically derived bacterial strains isolated in both metropolitan and regional hospitals (Table 3). These included *P. aeruginosa* AG1 (2010 isolate), two additional *P. aeruginosa* strains (RA and RB) known to harbor *IMP-18* based on local WGS analysis, and 116 isolates from an ongoing research project on carbapenem-resistant bacteria. The tailored qPCR assay identified 14 (11.8%) isolates as positive for *IMP*, which were negative by the automated qPCR. Among these, 11 were also *VIM*-positive. Amplification of the 16S rRNA gene was successful in all 119 cases. Ct values for the *IMP* gene ranged from 15.97 to 21.76, and for the *VIM* gene from 11.31 to 15.97 (Table 3 and Table 4). Species distribution included 12 *P. aeruginosa*, one *P. putida*, and one *Enterobacter cloacae* complex isolate. All positive strains were isolated from hospitals in the Metropolitan Area of Costa Rica.

Finally, all isolates with discordant results were further analyzed by MIC testing and whole-genome sequencing, as shown in Table 4. Phenotypic resistance to carbapenems (meropenem and imipenem) showed that all strains were resistant to imipenem, although two isolates (R60 and R86) were not resistant to meropenem. Whole-genome analysis confirmed the presence of the *IMP-18* gene in all strains. Additional *bla* genes were identified, and MLST revealed that *P. aeruginosa* strains belonged to either ST-111 (also harboring *VIM* as part of an integron) or ST-179 (without *VIM*). No ST could be assigned to *P. putida*, whereas the *E. cloacae* complex R36 was assigned to ST-90. For the *E. cloacae* complex, the isolate was reported with plasmids IncHI2/IncHI2A (327 bp and 630 bp, for both coverage 100% and identity 100%) and Col(pHAD28) (coverage 100% and identity 90.8%), but no bla genes were reported to be harbored within these elements.

Based on the whole-genome analysis, the tailored qPCR resulted in 100% sensitivity and specificity and positive and negative predictive values, unlike the automated qPCR, which had 0% sensitivity, 100% specificity, 88% accuracy, and negative predictive values.

## 4. Discussion

Carbapenems are essential antibiotics for treating nosocomial infections caused by Gram-negative bacteria such as Enterobacterales, *P. aeruginosa*, and *A. baumannii* [5]. However, the excessive or inappropriate use of carbapenems has contributed to the emergence and spread of carbapenemase-producing bacteria, which inactivate these antibiotics and limit therapeutic options [12]. Their detection requires specific phenotypic and molecular techniques, and their control demands strict epidemiological surveillance and effective infection prevention and control measures in clinical settings [8].

Automated molecular strategies for detecting antibiotic resistance genes, including carbapenem resistance, are a major advancement in modern clinical microbiology and one of the most important activities in diagnostic stewardship programs in clinical settings, supporting decision-making that impacts both patient care and public health [46]. In Costa Rica and Latin America, hospital-based circulation of carbapenem-resistant bacteria has been reported in several studies, including isolates carrying *IMP*, *VIM*, *NDM*, and *KPC* genes [23,24,26,32,47,48,49]. However, molecular tests, including automated qPCR assays, are often designed in other regions and may not include the alleles circulating in Latin America, sometimes leading to false-negative results [16,18,34]. This was the case addressed in the present study, in which a tailored qPCR test was implemented to extend the detection of *IMP* genes circulating in Costa Rica and Latin America. Although *IMP* has been identified in several Latin American countries, its prevalence is lower compared to other carbapenemases like *KPC*, *NDM*, and *VIM* [50,51].

In this study, the initial cases identified were those with negative results for *IMP* genes in automated qPCR, despite testing positive by immunochromatography. Up to this point, the *IMP* alleles were unknown, and it was unclear whether they differed from those reported in previous cases isolated more than ten years earlier. Using whole-genome sequencing, the alleles were identified as *IMP-18*. Thus, the newly designed primers could detect *IMP-18* and other additional alleles beyond those targeted by the commercial system. Although the primers used in the automated qPCR are unknown and this is a challenge for addressing false negatives in a commercial kit, the gene drop-out phenomenon explains the inconsistent results due to mutations and variations at the primer-binding sites [18], in this case for *IMP*, particularly for alleles such as *IMP-18* (present in all the positive cases). These variations would not necessarily affect the results of immunochromatographic tests, as the enzyme is detected via monoclonal antibodies binding to different regions of the (protein) sequence.

Although PCR-based detection is more sensitive for identifying resistance determinants, protein detection through molecular biology methods, such as immunochromatography, proved to be more conclusive and aligned more consistently with the phenotypic profile. This is a key finding that underscores the importance of using complementary laboratory techniques to validate results, including phenotypic testing, to enhance the study and surveillance of antibiotic resistance.

Furthermore, the inconsistencies reported came from isolates collected from various medical centers in Costa Rica. Whole-genome sequencing confirmed they were clones previously reported in Latin America [23,25,32,47], such as *P. aeruginosa* ST-111 (which also carries the *VIM* gene). These findings indicate that ST-111 clones continue to circulate 15 years after their first report, carrying both *IMP-18* and *VIM-2* genes [33]. As expected, in all ST-111 clones in this study, *VIM* was detected, but other ST clones lacking *VIM* harbored *IMP-18*. Thus, *VIM* detection (via qPCR or immunochromatography) could serve as a screening marker for ST-111 cases and help guide the reevaluation of negative *IMP* results, as found in this study.

Some automated systems focus on detecting specific imipenemases, for example, *IMP-1* [31,52]. The *IMP-1* allele was initially reported in Asian countries and is the predominant allele in some of these regions [53,54,55]. Interestingly, *IMP-1* has been scarcely reported in Latin America [56,57]. In contrast, *IMP-18* has been detected in several cases in Costa Rica, Mexico, Panama, Brazil, Puerto Rico, and other countries [23,25,32,47,48,58]. The first ST-111 case harboring both *IMP-18* and *VIM-2* was reported in *P. aeruginosa* AG1 by our research group in Costa Rica [33]. These genes are carried in class 1 integrons, which facilitate their horizontal spread among bacteria [13]. Moreover, in this study, although strains with inconsistent results have been identified in hospitals within the metropolitan area, many patients are transferred to and from regional hospitals. This inter-hospital mobility, if not strictly controlled, may contribute to the spread of high-risk clones [59], including strains with profiles like those observed in this study. Due to their multidrug resistance, genetic profile, and potential to cause nosocomial infections, identifying these bacteria is critical [5]. However, *IMP-18* was not detectable with the automated system.

This is an example of the consequences of the gene drop-out phenomenon, which impacts resistance gene detection and carries clinical and epidemiological consequences [17,18]. This concept gained relevance during the COVID-19 pandemic, when some multi-target PCR tests failed to detect one of the SARS-CoV-2 genes due to mutations [17]. For example, this occurred with the spike gene, a failure known as “S gene target failure (SGTF)”, and it enabled the indirect detection of variants like Delta and Omicron without immediate genomic sequencing [60]. Its occurrence prompted diagnostic updates and highlighted the importance of molecular biology in epidemiological surveillance and early variant detection.

In the context of antimicrobial resistance and diagnostic stewardship programs, gene drop-out in PCR can impact clinical practice by delaying diagnosis, hindering timely treatment, and compromising infection control in hospitals [16,61]. It may also lead to inappropriate antibiotic use and underestimation of resistance gene prevalence in surveillance studies [8,50].

From an epidemiological surveillance perspective, infections caused by carbapenem-resistant bacteria are a major public health problem, associated with high morbidity, mortality, and healthcare costs [1,62,63]. Therefore, carbapenems should be used under strict, targeted antibiotic stewardship guided by susceptibility testing and clinical guidelines [5]. Proper use is essential to preserve their efficacy and prevent the spread of resistance in Gram-negative pathogens [19]. However, in some cases, antibiotic use guidelines are based solely on molecular tests, which should be assessed to determine the reliability of detection results and whether adjustments are needed [64].

To minimize the gene drop-out problem, it is recommended to use multiplex PCR assays that amplify different gene regions, design degenerate primers, or include multiple probes covering known genetic variants [10,16,17,61]. In some cases, systems from different manufacturers are acquired, potentially using different primers. Additionally, as demonstrated in this study, complementing molecular findings with phenotypic or whole-genome sequencing techniques is useful [16,19]. Together, these strategies help prevent false negatives and ensure more reliable detection of antimicrobial resistance. Therefore, understanding the limitations and scope of molecular tests is essential for their implementation in clinical laboratories [8].

Moreover, this study is an example of effective collaboration between clinical laboratories and scientific research. An observation of inconsistent hospital-level results prompted a successful tailored assay for detecting additional *IMP* alleles that were likely undetectable by existing tests. Whole-genome sequencing played a key role in resolving the inconsistency and offering a diagnostic solution by tailored qPCR.

On the other hand, further microbiological and epidemiological analysis is expected to relate the isolates, identify other virulence and resistance determinants, and understand their genetic context. In the case of *P. aeruginosa*, the analyzed genomes are in line with previous reports (ST-111), as discussed. According to the local epidemiological surveillance system, carbapenem resistance in *P. aeruginosa* has varied between 9% and 16% in recent years for Costa Rica, which contrasts with the 30% for Latin America [65]. For the *E. cloacae* complex, the presence of *IMP* had been reported in an isolate in Costa Rica [66]; however, this new report also provides information on the specific allele *IMP-18*. In this bacterium, two plasmids were found, IncHI2/IncHI2A and Col(pHAD28), but *bla* genes were not reported as part of these elements, unlike other studies [67]. Further analyses will be conducted to study the evolutionary steps associated with these findings.

Jointly, this study supports genomic surveillance, outbreak detection, and monitoring of the evolution and spread of resistant clones locally and globally [19]. This knowledge can guide decision-making regarding protocols, best practices, and the acquisition of molecular techniques suited to local and regional needs [68]. This is particularly important during the current period of expansion and strengthening of molecular biology laboratories in healthcare centers worldwide following the COVID-19 pandemic [69]. That expansion was driven by the urgent need for rapid and accurate diagnostics using techniques like qPCR, along with increased public and private investment in healthcare infrastructure [70]. Additionally, the importance of genomic sequencing for variant detection and response to future health emergencies was recognized [71]. As a result, many hospitals now have enhanced capabilities for diagnosing infectious diseases, practicing personalized medicine, conducting clinical trials, modernizing healthcare systems, and training more professionals specialized in clinical molecular microbiology [69]. As in our case, the use of tailored qPCR to optimize molecular testing is cost-effective and feasible in labs with standard equipment; it requires minimal setup costs, basic molecular biology training, and reagents, and the quick turnaround makes it suitable for both research and diagnostics [68].

Finally, this study presents some limitations. First, the strain collection consists of carbapenem-resistant isolates and does not represent the broader population of bacteria causing infections. Also, only strains from Costa Rica were included, although these share resistance profiles with others reported in the Latin American region, including *P. aeruginosa* ST-111 harboring *IMP-18*. Amplification of alleles with variants in primer-binding sites was not assessed in this study, although these gene versions are not being reported in Latin America. We assessed the presence of carbapenemase genes, but not gene expression or functionality, nor the contribution of other mechanisms to the resistance phenotype. Lastly, while the inconsistencies reported involved negative qPCR results, immunochromatographic tests are also susceptible to failure, as previously reported [14], although such cases were not observed in this study.

## 5. Conclusions

This study highlights the diagnostic limitations of automated qPCR systems in detecting certain carbapenemase genes (including *IMP* alleles) circulating in Costa Rica and Latin America. The observed discrepancies between molecular and immunochromatographic results underscore the impact of the gene drop-out phenomenon, which can lead to false-negative results and hinder timely clinical and epidemiological responses. By using a tailored qPCR, we were able to detect previously unrecognized *IMP-18* alleles, demonstrating the value of adapting diagnostic tools to local genetic contexts. Sequencing results supported the effectiveness of the tailored molecular assay in detecting *IMP* genes and overcoming the *IMP* gene drop-out in a qPCR system for strains harboring resistance genes not common in other latitudes.

Our findings emphasize the importance of integrating phenotypic, molecular, and genomic approaches to ensure accurate detection of resistance genes. In the context of growing antimicrobial resistance and increasing molecular diagnostic capabilities, particularly following the COVID-19 pandemic, laboratories must remain aware of the limitations of laboratory tests and prioritize surveillance strategies tailored to regional needs. Moreover, collaborative efforts between clinical laboratories and research institutions, such as the one demonstrated here, are essential to strengthen diagnostic accuracy, outbreak detection, and infection control, thereby improving patient outcomes and guiding public health interventions.

## Figures and Tables

**Table 1:** Inconsistent results between laboratory tests to detect *IMP* gene in carbapenem-resistant strains.

Strain	Immuno-Chromatography	MBL Based on Automated qPCR	Whole-Genome Sequencing
*E. cloacae complex* R36	IMP-positive	*IMP*-negative	*IMP-18*-positive
*P. aeruginosa* R59	IMP-positive	*IMP*-negative	*IMP-18*-positive
*P. aeruginosa* AG1	Not tested	*IMP*-negative	*IMP-18*-positive

**Table 2:** Primers used for qPCR testing to extend the detection of *IMP* alleles, as well as *VIM* and 16S rRNA genes.

Gene	Name	Sequence	Length (pb)
*IMP*	IMP-F	GGAATAGAGTGGCTTAAYTCTC	22
	IMP-R	CCAAACYACTASGTTATCT	19
*VIM*	VIM-F	CGAAAAACACAGCGGCMCTTCT	22
	VIM-R	GTGGAGACTGCACGCGTTAC	20
16S rRNA	16S-F515	GTGYCAGCMGCCGCGGTAA	19
	16S-R926	CCGYCAATTYMTTTRAGTTT	20

**Table 3:** Screening of *IMP* alleles in 119 clinical carbapenem-resistant Gram-negative isolates using the tailored qPCR. *VIM* and 16S rRNA genes were included as complementary targets and internal controls, respectively. Ct values are based on a 0.100 threshold.

Study ID	Species	Reception Date	Hospital and Region	MBL-Automated qPCR *	Custom qPCR		
					Ct *IMP*	Ct *VIM*	16S rRNA
AG1	*P. aeruginosa*	2010	H1 metropolitan	*VIM*	16.9	13.36	+
RA	*P. aeruginosa*	2015	H1 metropolitan	*VIM*	17.57	14.23	+
RB	*P. aeruginosa*	2015	H1 metropolitan	*VIM*	16.36	13.95	+
R1	*K. pneumoniae*	dic-21	H7 metropolitan	*NDM*	-	-	+
R2	*A. baumannii*	jul-22	H7 metropolitan	*NDM*	-	-	+
R3	*S. fonticola*	jul-22	H7 metropolitan	*NDM*	-	-	+
R4	*P. putida*	nov-22	H1 metropolitan	*VIM*	19.01	12.65	+
R5	*S. marcescens*	sept-23	H1 metropolitan	*NDM*	-	-	+
R6	*K. pneumoniae*	sept-23	H1 metropolitan	*NDM*	-	-	+
R7	*K. pneumoniae*	sept-23	H1 metropolitan	*NDM*	-	-	+
R8	*E. coli*	sept-23	H1 metropolitan	*NDM*	-	-	+
R9	*P. mirabilis*	sept-23	H1 metropolitan	*NDM*	-	-	+
R10	*P. mirabilis*	sept-23	H1 metropolitan	*NDM*	-	-	+
R11	*S. marcescens*	sept-23	H1 metropolitan	*NDM*	-	-	+
R12	*P. aeruginosa*	sept-23	H1 metropolitan	*VIM*	19.62	15.57	+
R13	*K. pneumoniae*	sept-23	H1 metropolitan	*NDM*	-	-	+
R14	*K. pneumoniae*	sept-23	H1 metropolitan	*NDM*	-	-	+
R15	*K. pneumoniae*	sept-23	H1 metropolitan	*NDM*	-	-	+
R16	*P. aeruginosa*	oct-23	H1 metropolitan	*VIM*	15.99	13.46	+
R17	*P. aeruginosa*	oct-23	H1 metropolitan	*VIM*	15.97	15.97	+
R18	*A. baumannii*	oct-23	H1 metropolitan	*NDM*	-	-	+
R19	*K. pneumoniae*	dic-23	H7 metropolitan	No MBL	-	-	+
R20	*K. pneumoniae*	ene-24	H2 regional	No MBL	-	-	+
R21	*M. morganii*	ene-24	H2 regional	No MBL	-	-	+
R22	*E. coli*	ene-24	H2 regional	No MBL	-	-	+
R23	*E. coli*	ene-24	H2 regional	No MBL	-	-	+
R24	*E. cloacae complex*	ene-24	H2 regional	No MBL	-	-	+
R25	*P. mirabilis*	ene-24	H2 regional	No MBL	-	-	+
R26	*E. coli*	ene-24	H2 regional	No MBL	-	-	+
R27	*E. coli*	ene-24	H2 regional	No MBL	-	-	+
R28	*P. aeruginosa*	feb-24	H2 regional	No MBL	-	-	+
R29	*E. coli*	feb-24	H2 regional	No MBL	-	-	+
R30	*E. coli*	feb-24	H2 regional	No MBL	-	-	+
R31	*K. pneumoniae*	feb-24	H2 regional	No MBL	-	-	+
R32	*K. pneumoniae*	feb-24	H2 regional	No MBL	-	-	+
R33	*E. cloacae*	mar-24	H2 regional	No MBL	-	-	+
R34	*P. aeruginosa*	jun-24	H3 metropolitan	No MBL	-	-	+
R35	*P. aeruginosa*	jun-24	H3 metropolitan	No MBL	-	-	+
R36	*E. cloacae complex*	jun-24	H4 metropolitan	No MBL (IC: IMP-pos)	18.24	-	+
R37	*S. marcescens*	jun-24	H4 metropolitan	*NDM*	-	-	+
R38	*C. youngae*	jun-24	H4 metropolitan	*NDM*	-	-	+
R39	*S. marcescens*	jun-24	H4 metropolitan	*NDM*	-	-	+
R40	*K. aerogenes*	jun-24	H4 metropolitan	*NDM*	-	-	+
R41	*E. coli*	jun-24	H4 metropolitan	*NDM*	-	-	+
R42	*E. coli*	jun-24	H4 metropolitan	*NDM*	-	-	+
R43	*C. freundii*	jun-24	H4 metropolitan	*NDM*	-	-	+
R44	*E. cloacae complex*	jun-24	H4 metropolitan	*NDM*	-	-	+
R45	*K. pneumoniae*	jun-24	H4 metropolitan	*NDM*	-	-	+
R46	*K. pneumoniae*	jun-24	H4 metropolitan	*NDM*	-	-	+
R47	*K. aerogenes*	jun-24	H4 metropolitan	*NDM*	-	-	+
R48	*K. pneumoniae*	jun-24	H4 metropolitan	*NDM*	-	-	+
R49	*K. aerogenes*	jun-24	H4 metropolitan	*NDM*	-	-	+
R50	*C. youngae*	jun-24	H4 metropolitan	*NDM*	-	-	+
R51	*E. coli*	jun-24	H4 metropolitan	*NDM*	-	-	+
R52	*K. aerogenes*	jun-24	H4 metropolitan	*NDM*	-	-	+
R53	*K. aerogenes*	jun-24	H4 metropolitan	*NDM*	-	-	+
R54	*K. aerogenes*	jun-24	H4 metropolitan	*NDM*	-	-	+
R55	*K. oxytoca*	jun-24	H4 metropolitan	*NDM*	-	-	+
R56	*K. aerogenes*	jun-24	H4 metropolitan	*NDM*	-	-	+
R57	*E. coli*	jun-24	H3 metropolitan	No MBL	-	-	+
R58	*P. mirabilis*	jun-24	H3 metropolitan	No MBL	-	-	+
R59	*P. aeruginosa*	jul-24	H5 metropolitan	No MBL (IC: IMP-pos)	18.16	-	+
R60	*P. aeruginosa*	jul-24	H5 metropolitan	NT	20.17	-	+
R61	*P. aeruginosa*	jul-24	H3 metropolitan	*VIM*	18.13	11.88	+
R62	*P. aeruginosa*	jul-24	H3 metropolitan	No MBL	-	-	+
R63	*P. aeruginosa*	jul-24	H3 metropolitan	*VIM*	-	-	+
R64	*K. pneumoniae*	ago-24	H6 metropolitan	NT	-	-	+
R65	*P. aeruginosa*	ago-24	H6 metropolitan	NT	-	-	+
R66	*P. aeruginosa*	ago-24	H6 metropolitan	NT	-	-	+
R67	*K. pneumoniae*	ago-24	H6 metropolitan	NT	-	-	+
R68	*Salmonella sp*	ago-24	H6 metropolitan	NT	-	-	+
R69	*K. pneumoniae*	ago-24	H6 metropolitan	NT	-	-	+
R70	*K. aerogenes*	ago-24	H6 metropolitan	NT	-	-	+
R71	*P. aeruginosa*	ago-24	H3 metropolitan	No MBL	-	-	+
R72	*P. aeruginosa*	sept-24	H3 metropolitan	No MBL	-	-	+
R73	*P. aeruginosa*	sept-24	H3 metropolitan	No MBL	-	-	+
R74	*P. aeruginosa*	sept-24	H3 metropolitan	No MBL	-	-	+
R75	*E. coli*	sept-24	H7 metropolitan	No MBL	-	-	+
R76	*E. aerogenes*	sept-24	H7 metropolitan	No MBL	-	-	+
R77	*E. coli*	sept-24	H7 metropolitan	*NDM*	-	-	+
R78	*E. cloacae complex*	oct-24	H7 metropolitan	No MBL	-	-	+
R79	*K. aerogenes*	oct-24	H7 metropolitan	No MBL	-	-	+
R80	*E. coli*	oct-24	H8 regional	No MBL	-	-	+
R81	*K. pneumoniae*	oct-24	H8 regional	*NDM*	-	-	+
R82	*S. marcescens*	oct-24	H5 metropolitan	*NDM*	-	-	+
R83	*S. marcescens*	oct-24	H5 metropolitan	*NDM*	-	-	+
R84	*E. coli*	oct-24	H8 regional	*NDM*	-	-	+
R85	*K. pneumoniae*	oct-24	H8 regional	*NDM*	-	-	+
R86	*P. aeruginosa*	nov-24	H7 metropolitan	*VIM* (IC: VIM and IMP-pos)	18.01	12.71	+
R87	*E. cloacae*	nov-24	H6 metropolitan	NT	-	-	+
R88	*P. aeruginosa*	nov-24	H6 metropolitan	NT	21.76	11.31	+
R89	*K. pneumoniae*	nov-24	H5 metropolitan	*NDM*	-	-	+
R90	*P. aeruginosa*	nov-24	H5 metropolitan	*VIM*	21.11	13.62	+
R91	*M. morganii*	nov-24	H8 regional	*NDM*	-	-	+
R92	*S. marcescens*	nov-24	H8 regional	*NDM*	-	-	+
R93	*K. pneumoniae*	nov-24	H8 regional	*NDM*	-	-	+
R94	*E. coli*	nov-24	H8 regional	*NDM*	-	-	+
R95	*E. coli*	nov-24	H5 metropolitan	*NDM*	-	-	+
R96	*K. pneumoniae*	nov-24	H5 metropolitan	*NDM*	-	-	+
R97	*C. freundii*	dic-24	H5 metropolitan	*NDM*	-	-	+
R98	*E. coli*	dic-24	H5 metropolitan	*NDM*	-	-	+
R99	*K. aerogenes*	ene-25	H7 metropolitan	No MBL	-	-	+
R100	*C. freundii*	ene-25	H7 metropolitan	*NDM*	-	-	+
R101	*E. aerogenes*	ene-25	H7 metropolitan	*NDM*	-	-	+
R102	*S. fonticola*	feb-25	H7 metropolitan	*NDM*	-	-	+
R103	*E. coli*	mar-25	H7 metropolitan	*NDM*	-	-	+
R104	*K. pneumoniae*	abr-25	H8 regional	*NDM*	-	-	+
R105	*K. pneumoniae*	abr-25	H8 regional	*NDM*	-	-	+
R106	*E. coli*	abr-25	H8 regional	*NDM*	-	-	+
R107	*C. freundii*	abr-25	H8 regional	*NDM*	-	-	+
R108	*K. pneumoniae*	abr-25	H8 regional	*NDM*	-	-	+
R109	*K. pneumoniae*	abr-25	H8 regional	*NDM*	-	-	+
R110	*A. baumannii*	may-25	H6 metropolitan	*NDM*	-	-	+
R111	*A. baumannii*	may-25	H6 metropolitan	*NDM*	-	-	+
R112	*A. baumannii*	may-25	H6 metropolitan	*NDM*	-	-	+
R113	*A. baumannii*	may-25	H6 metropolitan	*NDM*	-	-	+
R114	*A. baumannii*	may-25	H6 metropolitan	*NDM*	-	-	+
R115	*K. pneumoniae*	may-25	H6 metropolitan	NT	-	-	+
R116	*E. cloacae complex*	may-25	H8 regional	*NDM*	-	-	+

* NT: Not tested, IC: Immunochromatography.

**Table 4:** Phenotypic and genomic profiles of carbapenem-resistant isolates identified as *IMP*-positive by qPCR.

Isolates		Phenotypic Profile *				Genomic Profile			
Species (Strain/ID)	Hospital (H)	Meropenem		Imipenem		qPCR		Whole-Genome Sequencing Analyses	
		MIC (µg/mL)	Profile	MIC (µg/mL)	Profile	Ct *IMP*	Ct *VIM*	MLST	*bla* Genes
*P. aeruginosa* AG1	*H1 metropolitan*	≥16	R	≥16	R	16.90	13.36	ST-111	*IMP-18*, *VIM-2*, *OXA-2*, *OXA-395*, *PDC-55*
*P. aeruginosa* RA	*H1 metropolitan*	≥16	R	≥16	R	17.57	14.23	ST-111	*IMP-18*, *VIM-2*, *OXA-2*, *OXA-395*, *PDC-55*
*P. aeruginosa* RB	*H1 metropolitan*	≥16	R	≥16	R	16.36	13.95	ST-111	*IMP-18*, *VIM-62*, *OXA-2*, *OXA-395*, *PDC-55*
*P. putida* R4	*H1 metropolitan*	≥16	R	≥16	R	19.01	12.65	Unassigned	*IMP-18*, *VIM-2*
*P. aeruginosa* R12	*H1 metropolitan*	≥16	R	NT	NT	19.62	15.57	ST-111	*IMP-18*, *VIM-2*, *OXA-2*, *OXA-395*, *PDC-55*
*P. aeruginosa* R16	*H1 metropolitan*	≥16	R	≥16	R	15.99	13.46	ST-111	*IMP-18*, *VIM-2*, *OXA-2*, *OXA-395*, *PDC-55*
*P. aeruginosa* R17	*H1 metropolitan*	≥16	R	≥16	R	15.97	15.97	ST-111	*IMP-18*, *VIM-2*, *OXA-2*, *OXA-395*, *PDC-55*
*E. cloacae complex* R36	*H4 metropolitan*	8	R	≥16	R	18.24	-	ST-90	*IMP-18*, *OXA-2*, *SHV-12*
*P. aeruginosa* R59	*H5 metropolitan*	8	R	≥16	R	18.16	-	ST-179	*IMP-18*, *OXA-2*, *OXA-396*, *PDC-374*
*P. aeruginosa* R60	*H5 metropolitan*	2	S	≥16	R	20.17	-	ST-179	*IMP-18*, *OXA-2*, *OXA-396*, *PDC-374*
*P. aeruginosa* R61	*H3 metropolitan*	≥16	R	≥16	R	18.13	11.88	ST-111	*IMP-18*, *VIM-2*, *OXA-2*, *OXA-395*, *PDC-55*
*P. aeruginosa* R86	*H7 metropolitan*	4	I	≥16	R	18.01	12.71	ST-111	*IMP-18*, *VIM-2*, *OXA-2*, *OXA-395*, *PDC-55*
*P. aeruginosa* R88	*H6 metropolitan*	≥16	R	≥16	R	21.76	11.31	ST-111	*IMP-18*, *VIM-2*, *OXA-2*, *OXA-395*, *PDC-55*
*P. aeruginosa* R90	*H5 metropolitan*	≥16	R	≥16	R	21.11	13.62	ST-111	*IMP-18*, *VIM-2*, *OXA-2*, *OXA-395*, *PDC-55*

* NT: Not tested.

## Data Availability

All processed data supporting the findings of this study are available within the paper and its Supplementary Information. Raw data are available from the corresponding author upon reasonable request.

## Supplementary Materials

The following supporting information can be downloaded at https://www.mdpi.com/article/10.3390/antibiotics14080772/s1, File S1: Multiple sequence alignment of all *IMP* alleles and redesigned primers.

## Abbreviations

The following abbreviations are used in this manuscript:

IMP	Imipenemase IMP
PCR	Polymerase Chain Reaction
qPCR	Real-time PCR
